# Novel Advances in Qualitative Diagnostic Imaging for Decision Making in Multidisciplinary Treatment for Advanced Esophageal Cancer

**DOI:** 10.3390/jcm13020632

**Published:** 2024-01-22

**Authors:** Shinichi Okazumi, Gaku Ohira, Koichi Hayano, Tomoyoshi Aoyagi, Shunsuke Imanishi, Hisahiro Matsubara

**Affiliations:** 1 Department of Surgery, Toho University Sakura Medical Center, Chiba 285-8741, Japan; sokazumiyrsh@sakura.med.toho-u.ac.jp; 2 Frontier Surgery, Graduate School of Medicine, Chiba University, Chiba 260-8670, Japan; k-hayano@chiba-u.jp (K.H.); matsuhm@faculty.chiba-u.jp (H.M.)

**Keywords:** esophageal cancer, neoadjuvant therapy, perfusion-CT, diffusion-MRI, FDG-PET

## Abstract

**Background:** Recently, neoadjuvant therapy and the succeeding surgery for advanced esophageal cancer have been evaluated. In particular, the response to the therapy has been found to affect surgical outcomes, and thus a precise evaluation of treatment effect is important for this strategy. In this study, articles on qualitative diagnostic modalities to evaluate tumor activities were reviewed, and the diagnostic indices were examined. **Methods:** For prediction of the effect, perfusion CT and diffusion MRI were estimated. For the histological response evaluation, perfusion CT, diffusion-MRI, and FDG-PET were estimated. For downstaging evaluation of T4, tissue-selective image reconstruction using enhanced CT was estimated and diagnostic indices were reviewed. **Results:** The prediction of the effect using perfusion CT with ‘pre CRT blood flow’ and diffusion MRI with ‘pre CRT ADC value’; the estimation of the histological response using perfusion CT with ‘post CRT blood flow reduction, using diffusion MRI with ‘post CRT ADC increasing’, and using FDG-PET with ‘post CRT SUV reduction’; and the downstaging evaluation of T4 using CT image reconstruction with ‘fibrous changed layer’ were performed well, respectively. **Conclusions**: Qualitative imaging modalities for prediction or response evaluation of neoadjuvant therapy for progressive esophageal cancer were useful for the decision making of the treatment strategy of the multidisciplinary treatment.

## 1. Introduction

Recently, in the treatment of advanced esophageal cancer, evidence for combined-modality therapy has been obtained, and the usefulness of neoadjuvant therapy [1,2] and conversion surgery has been reported [3,4]. Neoadjuvant therapy is known to improve the results of surgery for advanced esophageal cancer. In particular, neoadjuvant chemoradiotherapy (NACRT) showed a better prognosis than chemotherapy [5,6,7,8]. When a histologically effective response is obtained, the presence of a good response has been shown to significantly affect the resection rate [9] and the long-term prognosis [10,11]; thus, precise evaluation has become more important for determining the treatment strategy.

On the other hand, if we could predict sensitivity before neoadjuvant therapy, the treatment plan could be made with higher certainty. Therefore, the diagnostic aims when considering the neoadjuvant strategy are predicting the response before initiating therapy, evaluating the response after therapy, and assessing R0 surgical resectability. 

Given this background, a qualitative diagnostic method to evaluate tumor activity would be useful. Tumors show many physiological or biochemical activities, such as increased perfusion with angiogenesis [12,13], decreased diffusion and heterogeneity with cellularity, stroma or necrosis, and increased glucose metabolism with proliferative activity. In recent years, blood flow evaluation using perfusion CT or dynamic contrast-enhanced (DCE) MRI, diffusion evaluation using diffusion MRI, glucose metabolism evaluation using FDG-PET, and tissue-selective contrast-enhanced CT reconstruction have been developed and reported as qualitative evaluations and as biomarkers that can be used to predict and evaluate treatment response [14]. The diagnostic procedures are independent of tumor shape and changes in metrics.

## 2. Material and Methods

**Figure 1:**
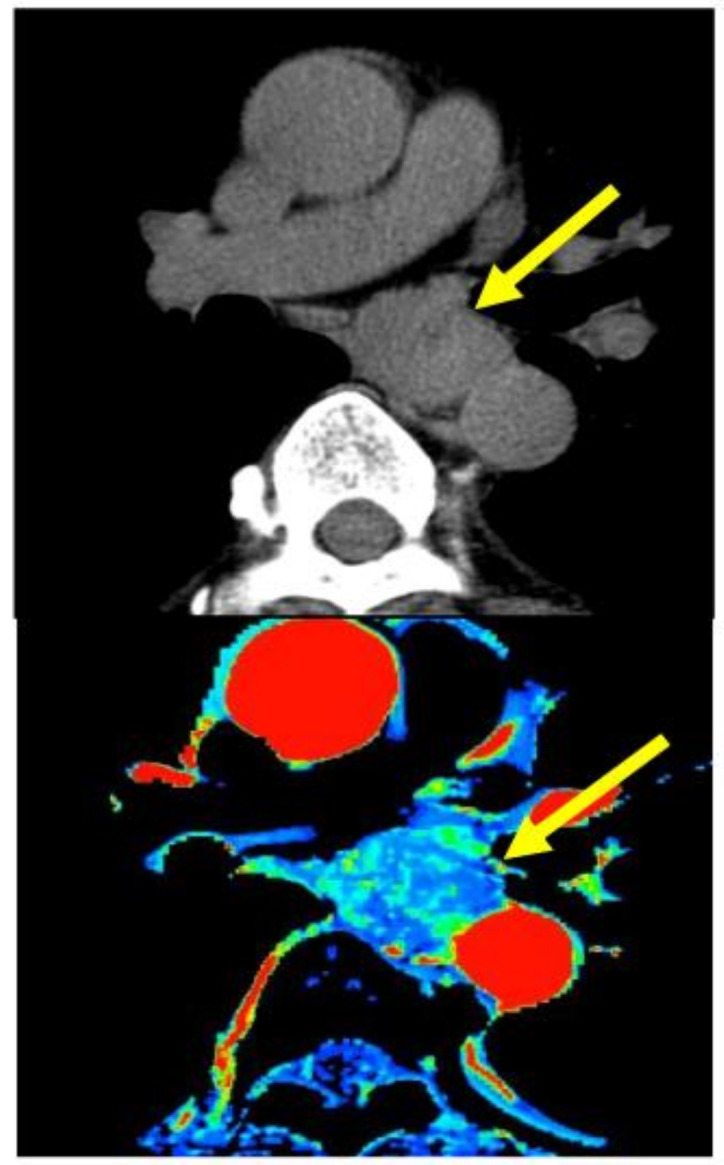
(**Upper**) plain CT image. (**Lower**) Calculated image using perfusion-CT for esophageal squamous cell carcinoma. Arrows indicate the esophageal tumor.

**Figure 2:**
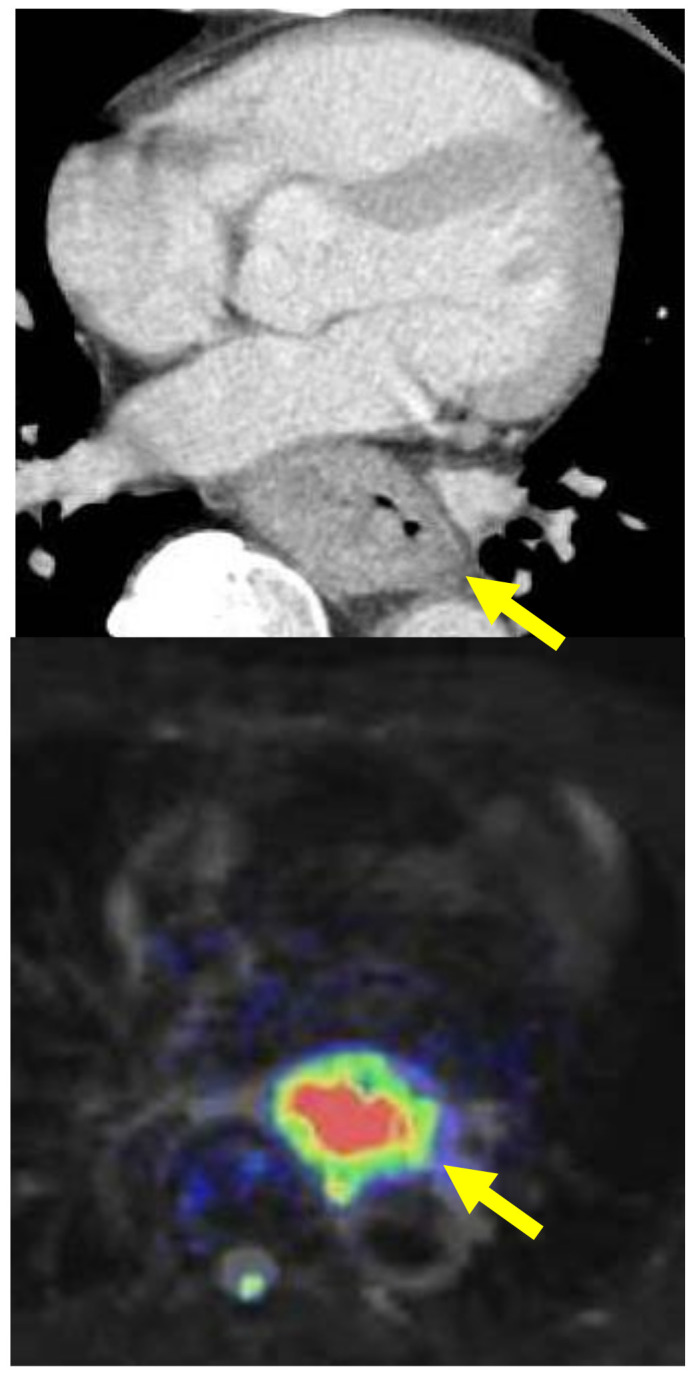
(**Upper**) Contrast-enhanced CT image. (**Lower**) Diffusion image obtained with 1.5T-MRI for esophageal squamous cell carcinoma. Arrows indicate the esophageal tumor.

**Figure 3:**
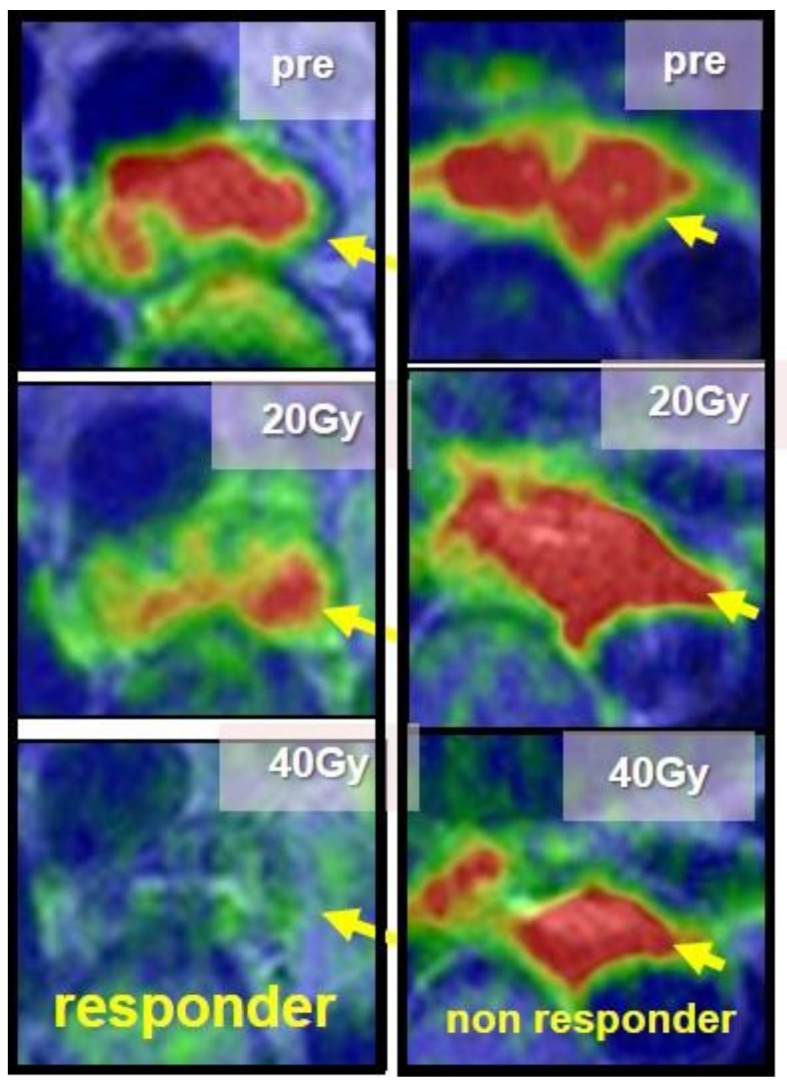
Comparison of the change in diffusion-MRI images of the tumor over the course of CRT for esophageal squamous cell carcinoma between responders and non-responders. Arrows indicate the tumor. Responders showed higher change in tumor diffusion than non-responders.

In this article, studies regarding these novel qualitative diagnostic procedures were reviewed. We searched papers on Pubmed with the key words ‘esophageal cancer’, ‘neoadjuvant chemoradiotherapy’, ‘diagnostic imaging’, and ‘response evaluation’ from 2006 to 2023, and we selected papers presenting results of comparisons between a diagnostic imaging evaluation method and histologic response investigated using surgical specimen after neoadjuvant treatment. And the future of qualitative diagnostic assessment for neo- adjuvant strategy was discussed according to the diagnostic aims, respectively. The criteria of ‘advanced cancer’ referred to tumor depth over T2, and that of ‘responder’ was of histological regression after neoadjuvant treatment over 50%. Our clinical images for the qualitative diagnosis of cases with advanced esophageal cancer during treatment are shown as examples in Figure 1, Figure 2 and Figure 3 and the methods of each imaging are described below.

Perfusion imaging: Using dynamic contrast-enhanced (DCE)-CT, the contrast medium (40 mL/body/8 s) was administrated and scanned using multi-detector CT (GE light speed ultra) continuously for 30 s at the tumor section and the tumor part perfusion was calculated with the apparatus. 

Diffusion imaging: Tumor diffusion was evaluated using the index of ADC (apparent diffusion coefficient) of the tumor region obtained at b = 1000 using 1.5T-MRI (Philips Achiva, Phlips Japan, Tokyo, Japan).

## 3. Results

### 3.1. Response Prediction before Neoadjuvant Therapy

A highly detailed prediction of treatment outcome requires evaluation of the physiological or biochemical characteristics of the tumor, which must be considered when selecting the drug delivery system or irradiation conditions. Tumors show angiogenesis with increased VEGF, which can be seen through evaluation of perfusion status, or stromal changes, which can be seen through evaluation of diffusion status [15]. Therefore, qualitative imaging modalities such as perfusion-CT or diffusion-MRI can show the dynamics of blood flow or diffusion of the tumor and surrounding tissue, which are considered useful for the prediction [16,17]. Table 1 shows the reported modalities and their indices for the prediction of the treatment effect. Most of the reported subjects were cases with esophageal squamous cell carcinoma. The usefulness of each respective modality is discussed below. 

***Perfusion analysis:*** It was thought that the high blood flow presented advantages with respect to pharmacokinetics or oxygenation to obtain a good pathological response. Perfusion-CT is a method that shows tumor tissue hemodynamics [18,19] (Figure 1). The tumor blood flow after CRT and the ◿blood flow could be evaluated using this modality. And the predictive ability of this method has also been reported. The quantity of tumor blood flow before treatment reflects the degree of response. Hayano et al. [20], Makari [21], and Li [22] reported that higher tumor blood flow could be the index for predicting responders. On the other hand, DCE-MRI also provides perfusion images. In the analysis of DCE-MRI, the Ktrans value is used as an index that reflects tissue permeability and blood flow. Lei et al. [23] and Ye et al. [24] reported that the pre-treatment Ktrans value was significantly higher in complete responders and could be a response predictor [25].

***Diffusion analysis:*** Tumors are considered to show trends in their diffusion status due to their microvessels and stromal characteristics [14]. Diffusion-MRI is a method to calculate the diffusion dynamics of tissue through imaging (Figure 2). According to the treatment effect, the apparent diffusion coefficient (ADC) is a quantitative measure of the state of diffusion. ADC value was reported to be inversely correlated to the tissue VEGF expression [14,26] or tumor stromal density [14]. Therefore, it could reflect the drug delivery or radiation circumstance and was expected to be a predictive marker of treatment response [26]. It has been reported in several articles that the diffusion of the tumor before treatment correlates with the degree of response to CRT [24,26,27,28]. Aoyagi et al. [27] described that the susceptibility to treatment could be predicted using this method, with ADC > 1.1 identifying responders, but no consensus was reached regarding the concept and index value for the prediction of response. Li et al. [29] speculated that tumors with high necrosis might have higher ADC values and that they would be associated with poor therapy outcomes. Hirata et al. reported that a high ADC value was related to a lower response [30]. 

**Table 1:** Articles on imaging biomarkers and their indices predicted the histological effect of neoadjuvant therapy before treatment for esophageal cancer.

Author	Year	Modality	Patients	Pathology	Indices
Hayano et al. [20]	2007	Perfusion-CT	31	SCC	high pre-CRT blood flow > 50 (mL/min/100 g)
Makari et al. [21]	2007	Perfusion-CT	55	SCC	high pre-CRT blood flow > 70 (mL/min/100 g)
Li [22]	2015	Perfusion-CT	55	SCC	high pre-CRT blood flow > 36.1 (mL/min/100 g)
Lei et al. [23]	2015	DCE-MRI	25	SCC	high pre-CRT Ktrans value
Ye et al. [24]	2018	DCE-MRI	237	AC, SCC	high pre-CRT Ktrans value
Aoyagi et al. [27]	2011	DW-MRI	80	SCC	high pre CRT ADC value > 1.1 (10^−3^ mm^2^/s)
Ye et al. [24]	2018	DW-MRI	237	AC, SCC	high pre-CRT ADC value
Guo et al. [28]	2018	DW-MRI	78	SCC	high pre-CRT ADC value
Cong et al. [26]	2019	DW-MRI	52	SCC	high pre-CRT ADC value
Hirata et al. [30]	2020	DW-MRI	58	SCC	low pre-CRT ADC value

DCE: dynamic contrast-enhanced MRI; DW: diffusion-weighted; ADC: apparent diffusion coefficient; SCC: squamous cell carcinoma; AC: adenocarcinoma.

### 3.2. Response Evaluation Reflecting the Degree of Pathological Regression after Treatment

In neoadjuvant therapy, when a histologically effective response was obtained, the prognosis after surgery was significantly better [31,32]. Therefore, it becomes essential to assess the degree of response after treatment accurately to determine whether to proceed with surgical resection. Tumor perfusion, diffusion, glucose metabolism, and tissue heterogeneity and their relationships have been found to change according to the histologic response to therapy. Table 2 shows the reported modalities and their indices for the evaluation of the treatment effect. The reported subjects were cases with esophageal squamous cell carcinoma or adenocarcinoma. Usefulness of respective modality is discussed below.

***Perfusion analysis:*** It has been reported that, on perfusion CT, the tumor part shows a significant blood flow decrease with treatment, and the images reflect the histologic response. Significant tumor blood flow decreases were seen in responders to neoadjuvant chemoradiotherapy compared with non-responders (Figure 1), and responders were diagnosed using this index of tumor blood flow decrease. Hayano et al. [33] reported an index of a 15% decrease in tumor blood flow after treatment, which reflected histological response, and Stefanovic et al. reported [34] a post-CRT blood flow < 30 (mL/min/100 g) as a response. 

***Diffusion analysis:*** A consensus was seen in several reports that tumor diffusion had been shown to have a tendency to increase after treatment according to the degree of the histologic response (Figure 3), and ◿ADC (pre–post) was a good marker which reflected histologic response [17,35,36,37,38]. In our results obtained via diffusion-weighted imaging with 1.5T-MRI for advanced esophageal cancer during the treatment of CRT, the responder group showed a significant earlier increase in the ADC value at the irradiation dose of 20 Gy than the non-responder group (Figure 3), and it seemed that an evaluation of the early treatment effect would be possible. Moreover, the possibility of its evaluation in the early treatment course was also recommended in several articles [35,37,39]. Imanishi et al. [35] reported that responders to CRT showed an ADC increase of 15% at 20 Gy and 40% at 40 Gy. Guo et al. [28], Vollenbrock et al. [40], and Borggreve [39] reported that the optimal timing for early prediction of the effect was 2 weeks after the beginning of treatment. Vollenbrock et al. [40] described that the ADC increase with treatment effect was due to the early effect of radiotherapy on the tumor microenvironment inducing tumor necrosis with a loss of cell membrane, resulting in an increase in the ADC. 

***Glucose metabolism analysis****:* FDG-PET is a technique for imaging the state of glucose metabolism, which reflects tumor proliferative activity, and the degree of glucose integration is assessed with the standardized uptake value (SUV) calculated from an image [41]. Evaluations of the mean uptake of the tumor and of total uptake are possible, and the histologic responses have been reported to correlate with the mean uptake [42,43]. As indices to identify responders, Higuchi et al. [44] reported post-treatment SUV < 2.5, and Swisher et al. [42] reported SUV < 3.1. Furthermore, through evaluation in the early treatment period, a prediction of the final effect was also considered possible. Otto et al. [45] assumed that a 35% decrease in accumulation two weeks after the start of chemotherapy for esophageal adenocarcinoma was a predictor of response. This occurred before the morphologic decrease in the tumor, which was thought to be due to decreased glucose metabolism prior to its morphologic decrease. In addition, the prognostic value of metastatic lymph node uptake after treatment, as well as the main tumor, was reported [46], and Yasuda et al. reported the prognostic value of residual lymph node uptake after treatment [47,48]. 

**Figure 4:**
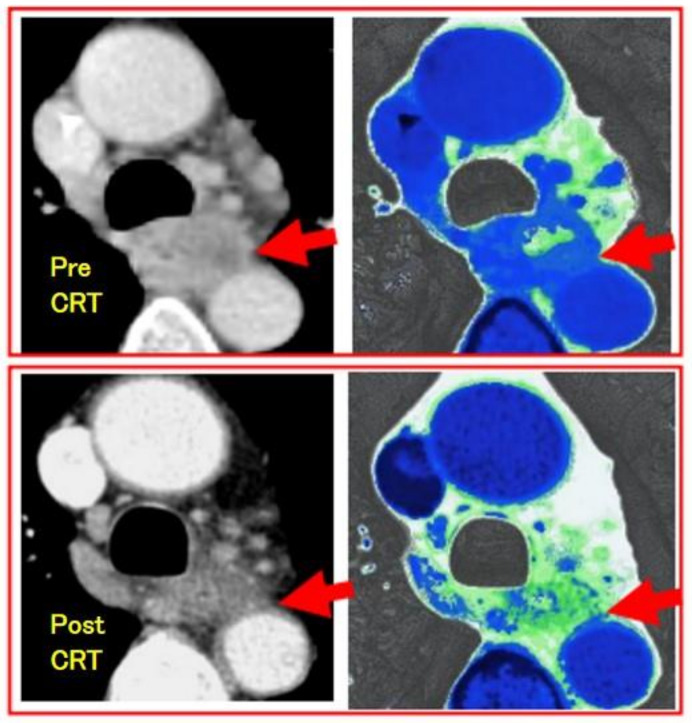
Downstaging evaluation using fibrous-tissue-enhanced CT reconstruction image of the case of esophageal squamous cell carcinoma after CRT. Contrast-enhanced CT was performed using multi-detector CT (GE light speed ultra). Arrows show the tumor. The upper is pre-CRT T4 image, which shows no interstitial plane between the tumor and aorta. The lower is post-CRT image, which shows fibrous plane as green and the case was diagnosed as downstaging and curatively resected with surgery.

***Heterogeneity analysis:*** Tumor heterogeneity has been reported to reflect malignancy or prognosis, and it has been studied through texture analysis using CT. It was reported that tumor texture showed a tendency to homogenize, decrease entropy, and increase uniformity after treatment [49,50,51,52]. When planning for conversion surgery, an evaluation of the heterogeneity of the effect on the tumor is needed to diagnose downstaging. If T4 status could be diagnosed as resolved, conversion surgery with R0 resection could be possible. For such a diagnostic aim, tissue-selective reconstruction of enhanced CT was applied to show the fibrous changes in the tumor due to treatment. In our study, contrast-enhanced CT (100 mL/body/30 s of contrast medium) was performed using multi-detector CT, and a depiction of the layer of connective tissue or adipose tissue between a tumor and adjacent organs was enabled through the contrast-enhanced CT image reconstruction method, which emphasizes the CT values of the tissue (Figure 4). It was found to be useful for the diagnosis of T4 cases. Furthermore, after treatment, the decrease in the CT level of the tumor has been found to correspond to the histologic response. In T4-diagnosed cases before treatment, the presence of a fibrotic change in the tumor and the emergence of a fibrous layer between the tumor and the adjacent organs after treatment have been reported to be evidence of downstaging that would permit conversion surgery with R0 resection [53].

**Table 2:** Articles of imaging biomarkers and their indices reflected the histological effect of neoadjuvant therapy after treatment for esophageal cancer.

Author	Year	Modality	Patients	Pathology	Indices
Evaluation after treatment					
Hayano et al. [33]	2014	Perfusion-CT	32	SCC	pre–post-CRT blood flow reduction > 15%
Stefanovic et al. [34]	2015	Perfusion-CT	40	SCC	post-CRT blood flow < 30 (ml/min/100 g)
Imanishi et al. [35]	2013	DW-MRI	27	SCC	◿ADC (>40%)
Li et al. [38]	2017	DW-MRI	28	SCC	◿ADC, post-CRT ADC
Cheng et al. [36]	2018	DW-MRI	236	AC, SCC	◿ADC, post-CRT ADC
Heethuis et al. [37]	2018	DW-MRI	45	AC, SCC	◿ADC (>75%)
Borggreve et al. [17]	2019	DW-MRI	69	AC, SCC	◿ADC
Swisher et al. [42]	2004	FDG-PET	103	AC, SCC	post-CRT SUV (<3.1)
Higuchi et al. [44]	2008	FDG-PET	50	SCC	post-CRT SUV (<2.5)
Izumi et al. [46]	2015	FDG-PET	73	SCC	post-CRT SUVmax with T/N ratio
Boerggreve et al. [17]	2019	FDG-PET	69	AC, SCC	◿SUV
Evaluation at the early treatment course					
Imanishi et al. [35]	2013	DW-MRI	27	SCC	◿ADC (>15%: 20Gy after beginning of CRT)
Guo et al. [28]	2018	DW-MRI	78	SCC, AC	◿ADC (2 w after beginning of CRT)
Vollenblock et al. [40]	2019	DW-MRI	516	AC, SCC	◿ADC (2 w after beginning of CRT)
Borggreve et al. [39]	2020	DW-MRI	24	AC, SCC	◿ADC (>36%: 2 w after beginning of CRT)
Ott et al. [45]	2006	FDG-PET	65	AC	◿SUV (>35%: 2 W after beginning of CRT)

ADC: apparent diffusion coefficient; SUV: standardized uptake value; SCC: squamous cell carcinoma; AC: adenocarcinoma; CRT: chemoradiotherapy.

## 4. Discussions

The literature on the novel qualitative imaging modalities for the evaluation or prediction of histological effects after neoadjuvant therapy for progressive esophageal cancer, as well as downstaging evaluation for conversion surgery, was reviewed. Several investigations of the indices of the various modalities have been performed (Table 3). Perfusion CT and diffusion-MRI appear useful for prediction and effect evaluation during neoadjuvant treatment. Perfusion CT, diffusion-MRI, and FDG-PET reflect the histologic response and are useful for precise evaluation. Heterogeneity evaluation using tissue-selected image reconstruction with contrast-enhanced CT can help in the diagnosis of downstaging after chemoradiation, which supports decision making in conversion surgery.

These modalities with the indices were useful for decision making in multidisciplinary treatment at every treatment stage, not only to choose the most effective method but also to avoid invasive and non-effective ones. The limitation is that there were few choices for strategy decision making for the cases that show poor effect prediction. In the future, alternative neoadjuvant therapy for them besides chemo radiation should be found, and then qualitative imaging modalities would be more useful.

## 5. Conclusions

Qualitative imaging modalities using indices of the respective modalities for the prediction or evaluation of histological effects after neoadjuvant therapy for progressive esophageal cancer were novel and useful techniques for making decisions regarding the treatment strategy for advanced esophageal cancer at several stages of the multidisciplinary treatment. In the future, even in the cases that show poor effect prediction, an alternative neoadjuvant therapy besides chemoradiation should be found, and then the qualitative imaging modalities would be more useful.

## Figures and Tables

**Table 3:** The diagnostic indices of qualitative imaging modalities for the evaluation of the effect of neoadjuvant therapy for advanced esophageal cancer.

	Perfusion CT	DCE-MRI	Diffusion-MRI	FDG-PET	Tissue Selected Rendering CE-CT
PreRP	high blood flow	high Ktrans value	high ADC value	(-)	(-)
DurRP	(-)	(-)	increase in ADC value	decrease in SUV	(-)
RE	decrease in tumor blood flow	◿Ktrans	increase in ADC value	decrease in SUV	decrease in CT value
DSE	(-)	(-)	(-)	(-)	appearance of fibrous changed layer

DCE-MRI: dynamic contrast-enhanced MRI; CE-CT: contrast-enhanced CT; ADC: apparent diffusion coefficient; PreRP: pretreatment responder prediction; DurRP: during treatment responder prediction; RE: response evaluation after treatment; DSE: downstaging evaluation for surgical resectability; FDG-PET: fluorodeoxyglucose-PET.

## Data Availability

The data used in this study are available on request from the corresponding author.
